# Unloosing the Gordian knot of peroxisome formation

**DOI:** 10.1016/j.ceb.2018.02.002

**Published:** 2018-02

**Authors:** Joseph L Costello, Michael Schrader

**Affiliations:** Biosciences, College of Life and Environmental Sciences, University of Exeter, Exeter EX4 4QD, United Kingdom

## Abstract

Peroxisome biogenesis is governed by molecular machineries, which are either unique to peroxisomes or are partially shared with mitochondria. As peroxisomes have important protective functions in the cell, modulation of their number is important for human health and disease. Significant progress has been made towards our understanding of the mechanisms of peroxisome formation, revealing a remarkable plasticity of the peroxisome biogenesis pathway. Here we discuss most recent findings with particular focus on peroxisome formation in mammalian cells.

**Current Opinion in Cell Biology** 2018, **50**:50–56

This review comes from a themed issue on **Cell architecture**

Edited by **Celeste M Nelson** and **Franck Perez**

For a complete overview see the Issue and the Editorial

Available online 21st February 2018

**https://doi.org/10.1016/j.ceb.2018.02.002**

0955-0674/© 2018 The Authors. Published by Elsevier Ltd. This is an open access article under the CC BY license (http://creativecommons.org/licenses/by/4.0/).

## Introduction

Peroxisomes are ubiquitous, single-membrane-bound, multifunctional organelles that play pivotal cooperative roles in the metabolism of cellular lipids and reactive oxygen species (ROS) and are essential for human health and development [1]. They show remarkable plasticity and responsiveness, constantly adapting their number, shape, position, and metabolic functions to changing physiological requirements. This requires dynamic processes which modulate peroxisome abundance by organelle formation (biogenesis), degradation (auto/pexophagy), or inheritance (cell division).

Peroxisome biogenesis disorders, which often combine loss of peroxisome function with altered peroxisome number and plasticity, are associated with developmental defects, neurodegeneration, eye problems, and hearing loss [2]. Peroxisome abundance and subsequent alterations in ROS levels have also been reported to influence neuronal firing and feeding behaviour in obese mice [3], or to protect sensory cells of the inner ear from sound exposure [4]. These findings highlight the importance of peroxisome plasticity and regulation of peroxisome number in health and disease and underline that peroxisomes, which contribute to ROS homeostasis, breakdown of toxic lipids and combat of pathogens, have important protective functions (Box 1).

Box 1 **Peroxisome biogenesis and function**

**Figure 1:**
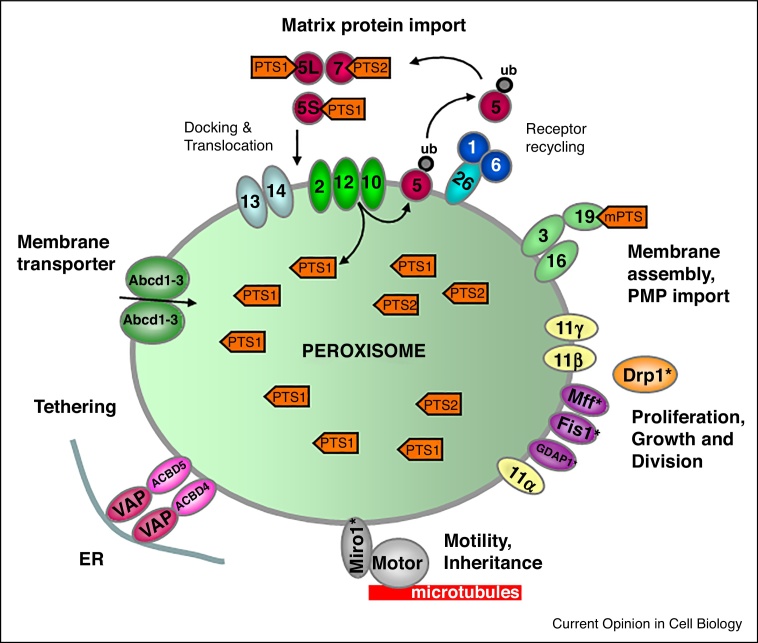
Schematic overview of the molecular machineries involved in the biogenesis of mammalian peroxisomes. **Matrix protein import:** After synthesis on free ribosomes, cargo proteins containing the peroxisomal targeting signals PTS1 or PTS2 bind to the corresponding cytosolic receptors Pex5 or Pex7 and form receptor-cargo complexes. The Pex7–cargo complex requires Pex5L, the long isoform of Pex5, for import. Import is achieved by a complex set of integral or peripheral PMPs that form the matrix protein import machinery, which mediates docking of the cargo-bound import receptor at the peroxisomal membrane, cargo translocation into the matrix of the organelle by a dynamic translocon, and export of the receptor back to the cytosol. Recycling of the receptor involves its ubiquitination (ub) and extraction from the membrane by an AAA-ATPase complex (Pex1, Pex6). **Membrane assembly and insertion of PMPs** (containing an mPTS) depends on Pex19, Pex3 and Pex16. Pex19 functions as a cycling receptor/chaperone, which binds the PMPs in the cytosol and interacts with Pex3 at the peroxisomal membrane. **Proliferation, growth and division**: Pex11α, Pex11β and Pex11γ are involved in the regulation of peroxisome size and number. Pex11β remodels the peroxisomal membrane, and interacts with the membrane adaptors Mff and Fis1, which recruit the dynamin-like fission GTPase Drp1 to peroxisomes, which is activated by Pex11β. **Motility and Inheritance**: Miro1 serves as membrane adaptor for the microtubule-dependent motor proteins kinesin and dynein [19•, 38••, 64••]. **Tethering**: ACBD5 and ACBD4 interact with ER-resident VAPA/B to mediate peroxisome-ER contacts. **Membrane transporter**: only the ABC transporter proteins involved in fatty acid uptake are shown. Proteins with a dual localisation to both peroxisomes and mitochondria are marked with an asterisk.

Peroxisome biogenesis requires the generation of a membrane and subsequent targeting and insertion of peroxisomal membrane proteins (PMPs) into the lipid bilayer, and import of soluble enzymes into the peroxisomal matrix [56, 57]. Unlike mitochondria, peroxisomes do not contain DNA or protein synthesis machinery, and have to import all soluble proteins post-translationally from the cytosol. This supported the concept of peroxisomes as autonomous organelles which receive all PMPs and matrix proteins from the cytosol [58]. Observations on ER-mediated targeting of PMPs and *de novo* formation challenged the classical view linking peroxisome biogenesis more closely to the ER. Matrix proteins and (most) PMPs are targeted through largely conserved, but distinct import machineries with unique properties (Figure 1). A hallmark is the import of fully folded or even oligomeric matrix proteins through a dynamic protein translocon [59]. The import machineries are composed of peroxins (Pex proteins), essential biogenesis factors, whose dysfunction can either block matrix protein import (resulting in empty peroxisomal membrane structures, so called ‘ghosts’) or membrane biogenesis and PMP import (e.g. loss of Pex3, Pex16, or Pex19 results in the absence of functional peroxisomes). As cells with Pex deficiencies are viable, they present ideal models to study peroxisome biogenesis. The core biogenic machinery of peroxisomes has been identified in yeast mutants and shown to be largely conserved across species. New peroxins are still discovered [26, 60, 61], and new roles in the combat of pathogens, cell fate decision and healthy ageing have been associated with peroxisomes [43, 46••, 62, 63]. Important functions of mammalian peroxisomes include the breakdown of fatty acids by peroxisomal β-oxidation (in cooperation with the mitochondrial β-oxidation pathway), the synthesis of bile acids in the liver, the decomposition of hydrogen peroxide by peroxisomal catalase, and the synthesis of ether-phospholipids (e.g. myelin sheath lipids) and docosahexaenoic acid (in cooperation with the ER). Mammalian peroxisomes also serve as important signalling platforms modulating physiological and pathological processes such as inflammation, apoptosis, cellular ageing, cancer development, immunity, and host–pathogen interactions.

The biogenesis of peroxisomes involves the formation of a peroxisomal membrane, the targeting and insertion of peroxisomal membrane proteins (PMPs), the import of soluble matrix proteins (Box 1), and the modulation of peroxisomal number, shape, and cellular position. These processes are governed by molecular machineries, which are either unique to peroxisomes or are partially shared with other organelles (Figure 1). In recent years, a number of unexpected observations in different model organisms have given new twists to the mechanisms of peroxisome formation, evolving concepts of indirect PMP targeting, ER-driven preperoxisomal vesicle formation and *de novo* formation of peroxisomes (reviewed in [5, 6, 7, 8]). This has added complexity to the current model of peroxisome formation, and the challenge ahead is to build an overall understanding of the general process. Here, we do not pretend to untie the Gordian knot of peroxisome formation, but will discuss most recent findings with particular emphasis on peroxisome formation in mammalian cells.

## Growth and division versus *de novo* formation

It is now generally accepted that peroxisome biogenesis can occur through both the classical route of growth and division of pre-existing organelles, or through an alternate route of *de novo* formation of nascent peroxisomes (reviewed in [5, 7]). Peroxisomal numbers are, however, primarily controlled by growth and division [9].

The *de novo* route of peroxisome formation was promoted by studies in yeast mutants lacking peroxisomes due to a loss of the membrane biogenesis factors Pex3 or Pex19. Remarkably, peroxisomes reappeared upon reintroduction of a functional copy of the gene. This led to a model where several key PMPs target the ER and sequester into preperoxisomal vesicles, which are released from the ER and can then form import-competent peroxisomes which grow and divide to multiply [10]. The initiation of *de novo* formation at the ER was, however, questioned, as preperoxisomal vesicles were also observed in yeast cells lacking Pex3 or Pex19; the vesicles had been overlooked because they were degraded by autophagy [11]. Studies in yeast have suggested a role for the reticulon-like proteins Pex30 and Pex31 in the generation of an ER subdomain in which preperoxisomal vesicles bud, supporting the ER origin of preperoxisomal vesicles [12•, 13, 14].

Although the ER-derived biogenic route is controversially discussed [5, 6, 7, 8], the ER is now recognized as an important contributor towards peroxisome biogenesis and peroxisomes are considered as semi-autonomous organelles, which depend on other organelles such as the ER to obtain lipids or even certain proteins [15]. The model of *de novo* biogenesis of peroxisomes has recently received another twist through studies using human fibroblasts from patients lacking the membrane biogenesis factors Pex3 or Pex16 which are devoid of peroxisomal membranes [16^••^]. When the missing peroxin was reintroduced, Pex3 targeted mitochondria where it exited in preperoxisomal vesicles. Pex16, however, trafficked to the ER and was released in vesicles that appeared to fuse with the mitochondria-derived preperoxisomes, thereby generating import competent, new peroxisomes. These findings point to a contribution of both ER and mitochondria to the *de novo* formation of peroxisomes in mammalian cells [16^••^].

Although PMPs in yeast lacking peroxisomes preferentially target the ER, a Pex3 fusion protein containing a mitochondrial targeting signal was routed to mitochondria in Pex3-deficient cells and induced *de novo* formation of mitochondria-derived import-competent peroxisomes. These findings suggest that mitochondria in yeast and mammalian cells can generate peroxisomes *de novo* when Pex3 targets mitochondria, and it was further hypothesized that natural or artificial targeting of Pex3 to any endomembrane may initiate peroxisome formation [17]. Therefore the key event in *de novo* formation may be the initial targeting of PMPs in the absence of peroxisomes.

## PMP targeting to multiple membranes

In contrast to yeast, many PMPs are routed to mitochondria in mammalian cells lacking peroxisomes. These include peroxins, but also membrane transporter and tail-anchored (TA) membrane proteins [18, 19•]. How these PMPs are inserted into the mitochondrial membrane is not well understood, but may involve the TOM machinery. Furthermore, peroxisomes and mitochondria in mammalian cells have an intimate, cooperative relationship, which includes dual targeting and sharing of proteins [20]. A recent study on the targeting of TA proteins revealed that a combination of TMD hydrophobicity and tail charge determines targeting to distinct organelles [19^•^]. As only subtle changes in the tail charge were required to shift TA protein targeting between peroxisomes and mitochondria, it is likely that advantageous protein exchange was driven through mutations in targeting regions altering binding affinities for targeting receptors such as Pex19 during co-evolution [21]. Overlap in targeting information may explain why so many PMPs are routed to mitochondria in mammalian cells when peroxisomal membranes are absent and/or the PMP import machinery is compromised. The targeting properties of PMPs can differ between organisms and species resulting in different affinities for organelle import receptors and chaperones, and may explain the preferential ER or mitochondrial localisation in yeast and mammalian cells. Binding affinities and the accessibility and abundance of import receptors may also influence direct or indirect targeting of PMPs under physiological conditions when peroxisomes are present [6, 8, 22]. For example, mammalian Pex3 targets directly to peroxisomes via Pex16/Pex19, as well as to the ER via a Pex16-dependent, Pex19-independent route [23, 24]. Peroxins may also fulfil additional functions at the ER, and a role for Pex19 and Pex3 in coordinated biogenesis of lipid droplets and peroxisomes has already been proposed [25].

A detailed understanding of targeting mechanisms, how proteins are routed to multiple membranes and how this is regulated becomes more important in light of the recent studies discussed above, regarding the role of the ER and mitochondria in peroxisome formation [12•, 16••]. As peroxisomal membrane biogenesis depends on only three proteins (a functional yeast homologue of Pex16 was recently identified [26]), the core proteins Pex3/Pex16/Pex19, once targeted, can exploit non-peroxisomal membranes for the generation of preperoxisomal structures delivering the initial membrane for *de novo* formation and subsequent recruitment of other PMPs. How this works, is not well understood, but roles for Pex3 and Pex19 in intra-ER sorting and budding have been revealed in yeast [27^•^] which may be supported by Pex30 and Pex31 [12^•^]. Specific proteins for a *de novo* pathway for peroxisomes have yet to be identified, and peroxins and PMPs appear to use existing machinery, for example, the ER translocon or GET complex to enter the ER.

**Figure 2:**
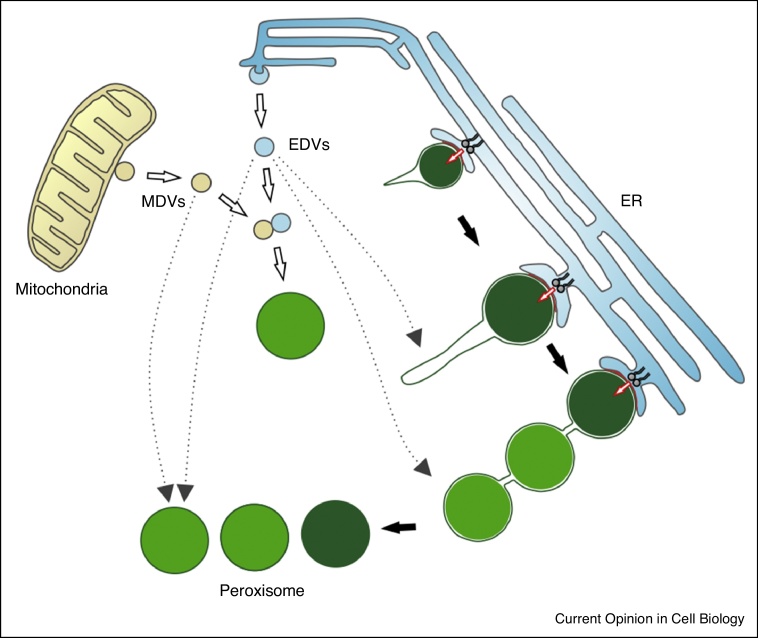
Schematic representation of mechanisms for peroxisome formation in mammalian cells. Peroxisome formation by **growth and division** follows a multistep maturation process involving peroxisomal membrane remodelling and elongation, membrane constriction and final scission. Membrane expansion requires peroxisome-ER contact (red line) and lipid transfer (red arrow), generating a membrane compartment which imports newly synthesised PMPs and matrix proteins. ***De novo* peroxisome formation**: In the absence of pre-existing peroxisomes, preperoxisomal vesicles can be generated at the ER (EDV) and mitochondria (MDV), which may fuse and mature into new import-competent peroxisomes. These newly formed peroxisomes will further multiply by growth and division. In the presence of peroxisomes, preperoxisomal vesicles may fuse with growing or existing peroxisomes to supply certain proteins and lipids. EDV, ER-derived vesicles; MDV, mitochondria-derived vesicles; newly formed peroxisomes are coloured in light green.

If the ER-derived and mitochondria-derived routes only contribute to *de novo* peroxisome formation or also have a more physiological role in replenishing existing peroxisomes with PMPs and lipids, remains to be established (Figure 2). An interesting aspect for future studies may be to investigate if these processes are linked to or driven by quality control mechanisms at organelle membranes to degrade or re-localise proteins with altered location. Mitochondria-derived vesicles, which target lysosomes for protein degradation or peroxisomes have been reported [28]. Preventing PMPs from aberrantly targeting mitochondria may be of particular relevance for mitochondrial function. In line with this, a quality control system for TA proteins mediated by the AAA ATPAse Msp1 in yeast (ATAD1 in mammals) has been reported, which allows identification and extraction of mis-localised proteins [29, 30]. Intriguingly, Msp1 and ATAD1 both localise to mitochondria and peroxisomes [30, 31] and it was suggested that protein selectivity could depend on organelle specific factors. Accordingly, Pex3 was shown to shield the TA protein Pex15 at peroxisomes inhibiting its removal, whilst at mitochondria Pex15 is not shielded by Pex3 and can be removed by Msp1 [32^•^]. If Pex3 protects peroxisomal TA proteins from Msp1-mediated degradation then targeting Pex3 to mitochondria could effectively override this process, allowing any mis-targeted peroxisomal TA protein to remain at mitochondria. Quality control mechanisms for PMPs may also exist at the ER which, together with self-organizational phenomena of PMPs and lipids, may drive the formation of preperoxisomal vesicles. As preperoxisomal vesicles are often degraded [11, 28], they may serve to route mis-localised PMPs to lysosomes or re-route them to peroxisomes.

## Shaping the peroxisomal membrane for division

The formation of peroxisomes by growth and division from pre-existing organelles requires remodelling and expansion of the peroxisomal membrane through the formation of tubular membrane extensions which then constrict and divide into new peroxisomes [33] (Figure 2). Multiplication by growth and division is an asymmetric process, which generates new peroxisomes via formation of a membrane compartment and subsequent import of newly synthesised matrix proteins [34, 35] (Figure 2). Several key proteins involved in peroxisome dynamics and multiplication have been identified (Figure 1), but their coordinated interplay, and how these processes are regulated is not well understood.

The membrane peroxin Pex11β, a remarkable key factor in the regulation of peroxisome abundance in mammals, has been linked to all stages of the growth and division process. Pex11β functions as a membrane-shaping protein, directly deforming and elongating the peroxisomal membrane before fission [35]. This activity depends on N-terminal amphipathic helices which interact with membrane lipids and on oligomerisation [36, 37]. Motor-driven pulling forces along cytoskeletal tracks can also contribute to membrane expansion (Figure 1) [38^••^]. Pex11β also supports the assembly of the fission machinery, which is composed of the dynamin-like GTPase Drp1 and the membrane adaptors Mff and Fis1 at the peroxisomal membrane (Figure 1) (reviewed in [33]). In addition, Pex11β functions as a GTPase activating protein for Drp1 during peroxisomal fission [39^•^]. Remarkably, several key fission proteins such as Drp1, Mff and Fis1 are shared with mitochondria, contributing to the ‘peroxisome-mitochondria connection’, which impacts on their cooperative functionality, contribution to diseases and promotes healthy lifespan [20, 40, 41, 42, 43]. Patients with a loss of Pex11β function have been identified [44, 45] and present with short stature, eye problems, progressive hearing loss and neurological defects. The metabolic functions of peroxisomes are either not or only slightly affected in patients with defects in peroxisome dynamics. This suggests that the symptoms relate to decreased peroxisome plasticity, underlining the importance of proper control of peroxisome abundance for cell performance. In line with this, altered peroxisome abundance in Pex11β-deficient epidermal cells resulted in abnormal mitosis and organelle inheritance, thus affecting cell fate decisions [46^••^]. Despite their fundamental importance to cell physiology, the mechanisms that mediate and regulate peroxisome membrane dynamics and abundance in humans are poorly understood.

## Peroxisome-ER tethering

Peroxisomes are not isolated entities but are a key part of the cells ‘social network’. They communicate and share signals, metabolites and proteins with other organelles. A recent study used multi-spectral imaging to simultaneously visualise six organelles and map their interactions [47^••^]. This allowed clear visualisation of the extent of interactions between peroxisomes and other organelles, in particular the ER and mitochondria. The molecular basis for the interactions between peroxisomes and mitochondria in mammalian cells remains unknown (although a genome-wide screening study in yeast identified a potential role for Pex11 [48]), but two studies independently identified peroxisomal ACBD5 and the ER protein VAP, as the missing factors which interact to form ER-peroxisome contact sites (Figure 1, Figure 2). The ACBD5-VAP hub not only plays a role in metabolite sharing/plasmalogen biosynthesis but also controls peroxisomal movement and membrane expansion [49••, 50••]. Ultrastructural analysis of cultured mammalian cells revealed an interaction of 70–80% of the peroxisomes with the ER. This may explain why only a small population of peroxisomes is observed to move in a microtubule-dependent manner in mammalian cells; indeed, loss of ACBD5 increases peroxisome motility providing a new role for a peroxisome-ER tether in the regulation of peroxisome movement in mammals.

Expansion and growth of the peroxisomal membrane requires lipids which are likely provided by the ER in a non-vesicular pathway [51]. Defects in peroxisome division (e.g. loss of Mff or Drp1) result in highly elongated peroxisomes, indicating a constant transfer of lipids from the ER to peroxisomes. As loss of peroxisome-ER interaction was shown to reduce membrane expansion of elongated peroxisomes, this supports a role of peroxisome-ER contacts in lipid transfer for peroxisome biogenesis. How lipids are transferred needs to be addressed in future studies. However, these observations may question a major role for ER-derived preperoxisomal vesicles in lipid transport to peroxisomes.

Mutations in ACBD5 and VAPB have both been linked to retinal dystrophy and white matter disease [52, 53] and amyotrophic lateral sclerosis [54], suggesting a possible link between loss of contact sites and cell dysfunction. By analogy to mitochondria-ER contacts, which involve a number of different complexes, there are likely to be other peroxisome-ER tethering complexes. In line with this a second potential peroxisomal tethering factor ACBD4, which also interacts with VAPB, has been identified [55].

## Concluding remarks

What is now clear is that the ER does play an essential role in generating new peroxisomes either via direct interaction at membrane contact sites and providing lipids for expansion of the peroxisomal membrane, allowing proliferation by growth and division, or by generating preperoxisomal vesicles which under certain conditions mature into new peroxisomes. Many key questions remain: how do PMPs enter and leave the ER and mitochondria? How do ER-derived and mitochondria-derived vesicles fuse and mature and how are they delivered to existing peroxisomes? What is the prevalence of the *de novo* pathway, what specific factors mediate it? How are lipids transferred between the ER and peroxisomes at contact sites? Despite the current entanglement, the global principle of peroxisome formation may be simpler than anticipated, and the Gordian knot of peroxisome biogenesis has yet to find its Alexander the Great.

## References and recommended reading

Papers of particular interest, published within the period of review, have been highlighted as:

• of special interest
•• of outstanding interest
